# Reply to the Letter to the Editor: The role of prompt engineering and referral completeness in evaluating GPT-4 for MRI protocols

**DOI:** 10.1007/s00330-025-12152-5

**Published:** 2025-11-14

**Authors:** Robert Terzis, Kenan Kaya, Lenhard Pennig

**Affiliations:** Institute for Diagnostic and Interventional Radiology, Faculty of Medicine and University Hospital Cologne, University of Cologne, Cologne, Germany

We thank Büyüktoka and Salbas for their careful reading of our work and for their constructive comments regarding the wording we used to describe our prompting strategy and our decision to exclude radiology request forms (RRFs) with insufficient clinical information. Their letter raises important points about reproducibility and external validity in studies of large language models (LLMs) in radiology.

Prompt strategy and engineering are key parts of studies evaluating the performance of LLM in radiology. The growing literature indicates that prompt formulation materially affects model behaviour and accuracy in clinical tasks [1]. We agree that our methods could have been clearer in distinguishing prompt development from inference. As described in our article, we refined a single instruction on four representative RRFs through 12 iterations, verified prompt stability by re-running those four RRFs five times each, and then applied the finalized prompt unchanged to each of the 100 test RRFs in independent chats [2]. Hence, our study reflects engineered prompting with zero-shot inference and without any in-context exemplars. To avoid ambiguity going forward, we will adopt this phrasing and explicitly separate prompt calibration on a small development subset (excluded from the test set) from the test-time inference procedure.

Further, we appreciate the observation of performance heterogeneity across subspecialties and agree that the comparatively weaker results in musculoskeletal imaging constitute an important finding. We respectfully disagree, however, that enhanced prompt engineering alone would have eliminated this effect. The scope of our study was to let Generative Pre-trained Transformer 4 (GPT-4) and radiology residents create sequence-level MRI protocols from scratch, solely based on the information included in the RRF. This more accurately reflects real-world clinical practice, where identification of suspected pathologies and guideline selection are essential elements of radiologic decision-making. Notably, guideline uniformity is least established in musculoskeletal imaging: the American College of Radiology and the European Society of Skeletal Radiology publish differing sequence parameters, and local practice patterns further modulate recommendations [3, 4]. This was specifically noted by our expert consensus in multiple GPT-4-generated musculoskeletal MRI protocols, especially in knee imaging. As we aspired to achieve findings with impact for the broader radiologic community, providing a specific, locally approved guideline to the LLM would have mitigated the generalizability of our results. Eventually, the key finding of varying performance of GPT-4 due to guideline inconsistencies would have been omitted. However, we concur that guideline-aware or few-shot prompt variants are logical next steps in less standardized areas. This would allow to specifically tailor generally functional prompts to distinct local preferences, available equipment and software limitations.

We also acknowledge that ambiguous inputs can degrade large language model performance [5]. Precisely for this reason, RRFs lacking sufficient clinical detail were excluded. Our primary objective was to determine whether GPT-4 can generate clinically applicable, sequence-level MRI protocols from the clinical history and question as stated in the RRF. To our knowledge at the time, no prior work had examined sequence-level protocol generation from scratch using GPT-4 across different radiological subspecialties. Including inadequate referrals would have introduced a major confound and biased the evaluation toward input quality rather than model capability. Importantly, identifying missing information and communicating with referring physicians remain human-facing tasks central to the radiologist’s role. In our discussion, we explicitly elaborated that the radiologist remains essential and that human interaction is fundamental to delivering perceptive and empathetic care. Further, we proposed future research to determine whether an LLM could first detect insufficient RRFs to streamline escalation, precisely the extension that Büyüktoka et al suggest [2]. Recent studies proposing the Reason for Exam Imaging Reporting and Data System (RI-RADS) indeed indicate that a large fraction of referrals are graded as incomplete or of poor quality, and that RI-RADS can reliably stratify referral completeness [6, 7]. We view this as a two-stage workflow worth studying: an upstream triage that flags inadequate referrals and, only when adequate, a downstream sequence-level protocol generation. Evidence from RI-RADS and CT-referral studies offers practical frameworks and ground truth for designing such experiments [5, 6].

In summary, we appreciate the opportunity to clarify that our study employed engineered prompting with zero-shot inference, and we will use this terminology henceforth to improve reproducibility. We also agree that evaluating LLMs on underspecified referrals is an important next step. As already emphasized in our article, we deem that radiologists remain central to patient care and propose future work on LLM-based detection of insufficient RRFs to optimize workflow.
